# Lateral Flow Immunoassay Coupled with Copper Enhancement for Rapid and Sensitive SARS-CoV-2 Nucleocapsid Protein Detection

**DOI:** 10.3390/bios12010013

**Published:** 2021-12-29

**Authors:** Tao Peng, Xueshima Jiao, Zhanwei Liang, Hongwei Zhao, Yang Zhao, Jie Xie, You Jiang, Xiaoping Yu, Xiang Fang, Xinhua Dai

**Affiliations:** 1 Technology Innovation Center of Mass Spectrometry for State Market Regulation, Center for Advanced Measurement Science, National Institute of Metrology, Beijing 100029, China; pengtao@nim.ac.cn (T.P.); s20090710020@cjlu.edu.cn (X.J.); s20090710033@cjlu.edu.cn (Z.L.); zhaoy@nim.ac.cn (Y.Z.); xiejie@nim.ac.cn (J.X.); jiangyou@nim.ac.cn (Y.J.); fangxaing@nim.ac.cn (X.F.); 2 College of Life Sciences, China Jiliang University, Hangzhou 310018, China; yxp@cjlu.edu.cn; 3 College of Ecology and Environment, Hainan University, Haikou 570228, China; hwzhao@hainanu.edu.cn

**Keywords:** SARS-CoV-2, nucleocapsid protein, signal amplification, copper deposition

## Abstract

The coronavirus disease 2019 (COVID-19) pandemic caused by severe acute respiratory coronavirus 2 (SARS-CoV-2) is still raging all over the world. Hence, the rapid and sensitive screening of the suspected population is in high demand. The nucleocapsid protein (NP) of SARS-CoV-2 has been selected as an ideal marker for viral antigen detection. This study describes a lateral flow immunoassay (LFIA) based on colloidal gold nanoparticles for rapid NP antigen detection, in which sensitivity was improved through copper deposition-induced signal amplification. The detection sensitivity of the developed LFIA for NP antigen detection (using certified reference materials) under the optimized parameters was 0.01 μg/mL and was promoted by three orders of magnitude to 10 pg/mL after copper deposition signal amplification. The LFIA coupled with the copper enhancement technique has many merits such as low cost, high efficiency, and high sensitivity. It provides an effective approach to the rapid screening, diagnosis, and monitoring of the suspected population in the COVID-19 outbreak.

## 1. Introduction

The coronavirus disease 2019 (COVID-19) pandemic caused by severe acute respiratory coronavirus 2 (SARS-CoV-2) has spread to 216 countries, and the cumulative number of confirmed cases has exceeded 200 million around the world. Some vaccines have been developed and administered, but the cumulative number of confirmed cases continues to increase every day. Thus, rapid screening, early detection, and timely diagnosis are still the main measures to prevent and control SARS-CoV-2 transmission. Rapid antibody detection was used as the supplementary means of nucleic acid detection for COVID-19 diagnosis before 2021 [1,2,3]. However, antibody detection has become meaningless for the screening of suspected populations since the beginning of vaccination against COVID-19. Nucleic acid detection is considered the gold standard, but its use is limited because of the time-consuming process, relatively high cost, and high professional and equipment requirements [4]. Hence, direct, rapid, point-of-care viral antigen detection methods without pretreatment are highly needed, especially in countries with serious outbreaks.

Coronavirus particles contain four structural proteins, namely, the nucleocapsid protein (NP), envelope protein, membrane protein, and spike protein [5]. Among them, the NP has been considered an ideal target for early diagnosis since the severe acute respiratory syndrome (SARS) outbreak in 2003 because the NP is predominantly and profusely expressed by severe acute respiratory syndrome coronavirus (SARS-CoV) [6]. The NP antigen can be detected up to 1 day before the appearance of clinical symptoms; thus, NP is considered one of the best markers for SARS-CoV detection [7]. SARS-CoV-2 has high genetic similarity to SARS-CoV [8]; therefore, theoretically, the NP antigen of SARS-CoV-2 can also be used as the diagnostic marker for COVID-19.

Mass spectrometry assays [9], electrochemical immunosensor assays [10,11], and lateral flow immunoassays (LFIAs) [12] have been developed for the detection of the SARS-CoV-2 NP antigen. Immunoassays, which are based on the specific reactions between antigen and antibody, may be a good choice for NP antigen detection because of their simple, convenient, and quick process. The LFIA, which combines chromatography technology with conventional immunoassay and nanomaterials, is considered the most attractive point-of-care testing device because it exhibits several advantages, including simple technical requirements, rapid detection capability, portability, affordability, high detection accuracy, and high efficiency [13]. Recently, colloidal gold nanoparticles (GCNPs), latex beads, fluorescent microspheres, and quantum dots have been used as labels to develop LFIAs for the rapid and sensitive screening of the NP antigen. For instance, Kim et al. [14] developed a cellulose nanobead-based LFIA platform using NP-specific single-chain variable fragment-crystallizable fragment fusion antibodies; Nichols’ group [12] described a half-strip LFIA with latex beads as the indicator, and Diao et al. [15] developed a fluorescence LFIA to rapidly detect the SARS-CoV-2 NP antigen in the laboratory. In addition, several antigen detection kits based on LFIAs have been approved by the National Medical Products Administration and marked as “Conformité Européenne”. However, the sensitivity of antigen detection was unsatisfactory compared with that of the reverse transcription-polymerase chain reaction assay [16,17]. Hence, the promotion of LFIA sensitivity is key for the rapid detection of SARS-CoV-2 NP antigen.

Wang’s group have applied a high-performance quantum dot nanobead [18] and magnetic quantum dot with a triple quantum dot shell [19] as novel labels to improve the sensitivity of the LFIA and accurately diagnose SARS-CoV-2; both labels exhibited good performances with sensitivity of 5.0 and 0.5 pg/mL in NP antigen detection, respectively. In addition, a robust Co–Fe@hemin-peroxidase nanozyme was used to amplify the immune reaction signal of a chemiluminescence paper assay with high sensitivity [20]. At present, GCNPs are the most common and popular signal indicators in LFIA because of their extraordinary physicochemical properties, such as good optical vision and high stability. Many strategies, such as silver staining, double labelling, enzyme catalysis, and a biotin-avidin system, have been applied to promote the sensitivity of GCNP-based LFIAs [21]. In addition to these strategies, GCNP-induced copper deposition can also enhance the LFIA signal and has the advantages of low cost, safety, and easy storage. Theoretically, Cu^2+^ is reduced into Cu^+^ in the presence of ascorbic acid, and then Cu^+^ is converted into Cu, which is deposited with the assistance of GCNPs and remarkably enhances the optical signal intensity of the LFIA. Liu’s group has taken advantage of this feature to improve the sensitivity of the colorimetric immunoassay and LFIA, which exhibited good performances in rapid detection [22,23,24].

**Figure 1:**
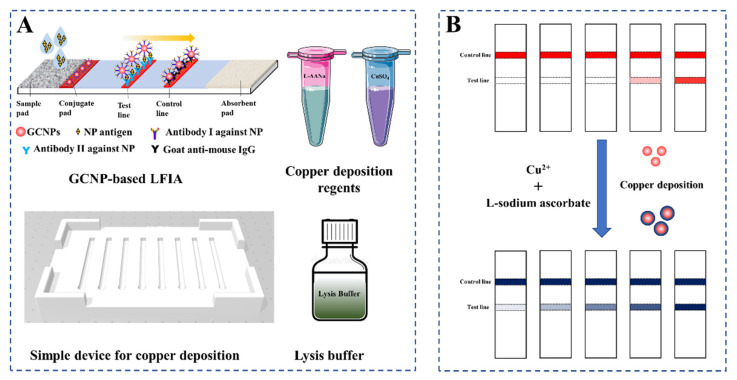
The diagram of GCNP-based LFIA coupled with copper deposition-introduced signal amplification for NP antigen detection. (**A**) Elements of the developed NP antigen detection system. (**B**) Results of antigen detection and signal amplification.

In this work, a GCNP-based LFIA coupled with copper deposition-induced signal amplification has been developed for rapid SARS-CoV-2 NP antigen detection. As shown in the scheme in Figure 1A, the developed NP antigen detection system includes a GCNP-based LFIA test strip, copper deposition, a simple homemade device for signal amplification, and a lysis buffer. In theory, the LFIA for NP antigen detection was based on the sandwich mode. Briefly, the test line without a red band indicates that the sample is negative for the antigen; the red band appears in the presence of the NP antigen, and the signal intensity of the test line is positively correlated with the NP antigen concentration. First, the LFIA test strips were immersed in CuSO_4_ solution. Then, the Cu^2+^ ions were reduced into Cu^+^ ions with the addition of L-sodium ascorbate (L-AANa) solution. Finally, with the assistance of the GCNPs captured on the test and control lines, the Cu^+^ ions were converted into Cu and deposited on the surface of GCNPs, which remarkably enhanced the visual signal intensity. Consequently, the sensitivity of LFIA for NP antigen detection improved considerably (Figure 1B). The developed direct, rapid point-of-care detection method for the SARS-CoV-2 NP antigen via copper deposition signal amplification was predicted to be a convenient supplementary approach to rapidly and effectively screen the suspected population in the COVID-19 pandemic.

## 2. Materials and Methods

### 2.1. Reagents and Apparatus

Mouse monoclonal antibodies I (No. DA027) and II (No. CSB-MA33255A2m) against SARS-CoV-2 NP antigen were purchased from Shanghai Jin’an Biotechnology Co. Ltd. (Shanghai, China) and Wuhan Huamei Biotechnology Co. Ltd. (Wuhan, China), respectively. Goat anti-mouse IgG was purchased from Beijing Yongjia Venture Company (Beijing, China). The NP solution reference material for COVID-19 (Code: GBW(E)091097) was provided by the National Institute of Metrology (Beijing, China). Chloroauric acid (HAuCl_4_·3H_2_O) was purchased from Sigma–Aldrich Chemical Corporation (St. Louis, Mo, USA). Copper sulfate pentahydrate (CuSO_4_·5H_2_O), L-AANa, trisodium citrate, PEG_20000_, bovine serum albumin (BSA), and Tween-20 were purchased from Aladdin Reagent Co., Ltd. (Shanghai, China). Sample pad, glass-fiber membrane, PVC pad, and absorbent pad were obtained from Kinbio Tech Co., Ltd. (Shanghai, China). Nitrocellulose (NC) membrane was purchased from Sartorius (Gottingen, Germany). All solvents and other chemicals were of analytical reagent grade.

The XYZ 3D film spraying instrument, CNC cutting machine (CTS300), and microcomputer automatic cutting machine (ZQ2402) were supplied by Kinbio Tech Co., Ltd. (Shanghai, China). Ultrapure water was purified with Milli-Q system from Millipore Corp. (Bedford, MA, USA).

### 2.2. Preparation of GCNPs

GCNPs were prepared according to the reference [25]. Briefly, 1 mL of HAuCl_4_·3H_2_O solution (1%, *w*/*v*) was added to 99 mL of ultrapure water and heated to boil. Then, 1.8 mL of 1% (*w*/*v*) sodium citrate solution was added quickly under rapid stirring. After the solution turned clear red, the solution was heated for another 5 min. The GCNP solution was cooled to room temperature naturally and then stored at 4 °C.

### 2.3. Synthesis of GCNP-Antibody I Detection Probes

The GCNP solution was adjusted to pH 8.0 by adding 0.2 M K_2_CO_3_ and then stirred with a constant speed mixer. Mouse monoclonal antibody I against NP antigen was diluted with phosphate buffer and added drop by drop under rapid stirring. After 1 h, 1% (*w*/*v*) PEG_20000_ solution and 10% (*w*/*v*) BSA solution were added successively to block the spare sites on the GCNPs for 20 min. The mixture was centrifuged at 8000 rpm at 4 °C for 30 min to remove the unconjugated free antibodies. The supernatant was discarded, and the precipitate was resuspensed with 1.0 M Tris-HCl containing 0.5% polyvinylpyrrolidone K30, 10% sucrose, 1% BSA, and 0.05% ProClin300. Finally, the GCNP-antibody I detection probe solution was obtained and stored at 4 °C until use.

### 2.4. Preparation of LFIA Test Strips

The LFIA test strip was composed of a sample pad, a conjugated pad, a NC membrane, an absorbent paper, and an adhesive PVC bottom plate. The prepared GCNP-antibody I detection probe solution was sprayed on the conjugated pad using an XYZ 3D film spraying instrument at a spray rate of 3 μL/cm, and the pad was dried at 37 °C for 2 h with a vacuum dryer. Goat anti-mouse IgG and mouse monoclonal antibody II were dispensed onto the NC membrane as the control and test lines, respectively, and then dried at 37 °C for 16 h. The fabricated LFIA test strip was cut into 3.0 mm-wide strips, stored at room temperature, and kept dry.

### 2.5. Copper Deposition-Induced Signal Amplification on the LFIA Strip

The NP antigen solution reference material was diluted into a series of standard solutions at concentrations of 1.0, 1.0 × 10^−1^, 1.0 × 10^−2^, 1.0 × 10^−3^, 1.0 × 10^−4^, 1.0 × 10^−5^, and 1.0 × 10^−6^ μg/mL, and 10 μL of each solution was mixed with 80 μL of lysis buffer (0.4% SDS, 0.6% TritonX-100, and 0.4% PVP were dissolved in 1.0 M Tris-HCl, pH 8.0) for 3 min. Each liquid was added to the sample pad of the LFIA strips, and the results could be observed by the naked eye after 15 min. Then, the strips were placed into the homemade device with the copper enhancement solution (50 mM L-AANa and 50 Mm CuSO_4_), and the results were obtained after 3 min.

## 3. Results and Discussion

### 3.1. Validation of GCNP-Mediated Copper Deposition

**Figure 2:**
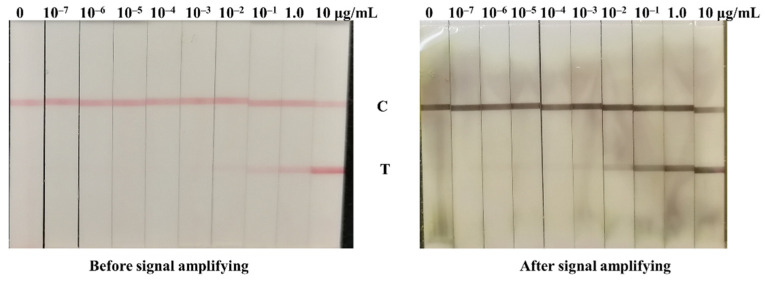
Comparison of the detection sensitivity before and after signal amplifying introduced by copper deposition.

The developed LFIA test strips were used to detect 0–10 μg/mL NP antigen to validate whether the signal was amplified by GCNP-mediated copper deposition. As shown in Figure 2, 0.1 μg/mL of the NP antigen could be distinguished from the negative before signal amplification. CuSO_4_ and L-AANa solutions with the same volume were added sequentially to the test strips. After 3 min, the red bands turned into black bands, and the LFIA strip for 0.01 μg/mL NP antigen detection also presented a clear band on the test line, which suggests that the detection sensitivity was successfully promoted by GCNP-mediated copper deposition.

### 3.2. Optimization of the Parameters

**Figure 3:**
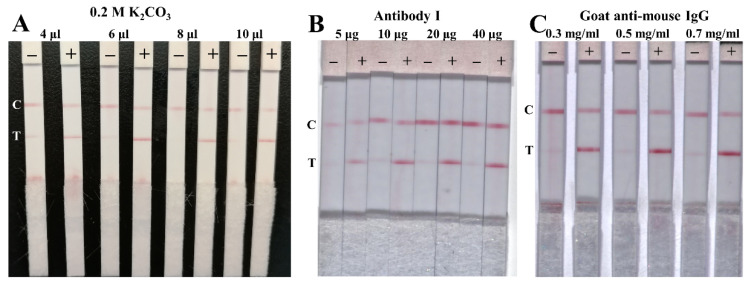
Optimization of the amount of 0.2 M K_2_CO_3_ solution used (**A**), antibody I used in the detection probes (**B**), and goat anti-mouse IgG on the control line (**C**). The positive sample was buffer spiked with 0.1 μg/mL of the NP antigen.

Six anti-NP antibodies were obtained from different companies and primarily evaluated by LFIA. Antibodies I and II from Shanghai Jin’an Biotechnology Co. Ltd. and Wuhan Huamei Biotechnology Co. Ltd. were selected in the present work (Figure S1). First, the pH and antibody amount of the GCNP-antibody I detection probe were optimized. Different volumes (4, 6, 8, and 10 μL) of 0.2 M K_2_CO_3_ solution were added to 1 mL of GCNP solution in order to adjust the pH of the detection probes. As shown in Figure 3A, the probes on the conjugated pad released completely, and the test line showed a clear red band without nonspecific adsorption when 8 μL of 0.2 M K_2_CO_3_ solution was added. Under the optimized pH, different amounts of antibody I (5, 10, 20, and 40 μg) was diluted with phosphate buffer, and then added into 1 mL of the GCNP solution. The prepared detection probes were evaluated with 0.1 μg/mL of the NP antigen; the result suggested that 10 μg of antibody I is the optimum amount (Figure 3B). Moreover, the UV-vis spectra of the GCNPs and detection probes (Figure S2) indicate that the characteristic absorption peak red shifted, which demonstrates that the antibody I conjugated with the GCNPs successfully. The amount of goat anti-mouse IgG coated on the control line seemed to have no significant influence on the detection result (Figure 3C); thus, 0.3 mg/mL of goat anti-mouse IgG was selected, in consideration of the cost.

**Figure 4:**
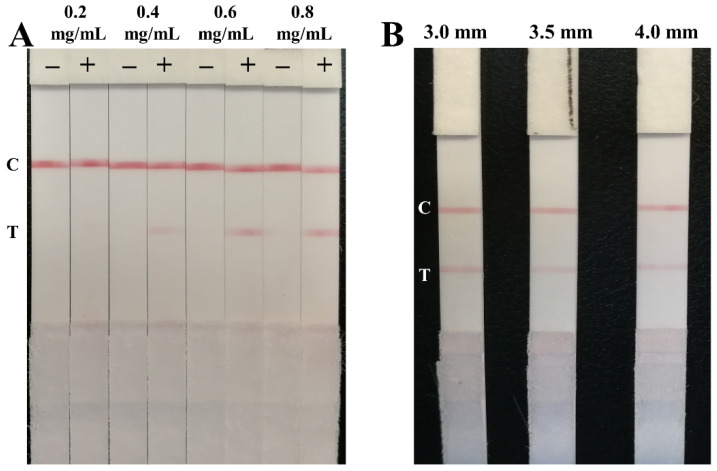
Optimization of the antibody II amount on the test line (**A**) and the width of LFIA test strip (**B**).

The sensitivity of the strip was affected by the amount of antibody coated on the test line. The mouse monoclonal antibody II against the NP antigen was diluted to 0.2, 0.4, 0.6, and 0.8 mg/mL with PBS buffer (0.01 M, pH = 7.4) and then sprayed on the NC membrane as the test lines. The positive sample with 0.1 μg/mL of the NP antigen was used to select the optimum amount of the antibody II. Based on the result in Figure 4A, 0.6 mg/mL of antibody II was selected. In addition, the liquid flow rate on the NC membrane is related to the width of the LFIA test strips, and a slower flow rate leads to a better immune reaction, which may directly impact the sensitivity of the LFIA. Hence, the fabricated LFIA was cut into 3.0, 3.5, and 4.0 mm widths to investigate the influence of test strip width on the sensitivity of the strip. The results in Figure 4B suggested that the color of the test line on the strip with a 3.0 mm width was the clearest. The results confirmed that the flow rate of liquid on the narrow test strip was the lowest, which increased the reaction time between the target and the detection probes and resulted in the improvement of the signal intensity.

### 3.3. Signal Amplification for NP Antigen Detection

**Figure 5:**
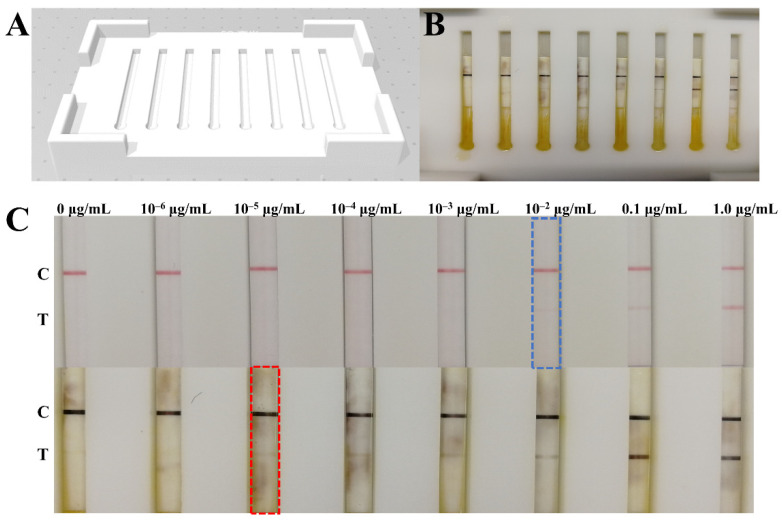
Simple device for copper deposition signal amplification (**A**,**B**); detection of NP antigen standard solutions before and after signal amplification (**C**).

First, a simple and portable device for copper deposition signal amplification was fabricated by 3D printing (Figure 5A). The signal amplification of each LFIA test strip can be carried out on separate trough (Figure 5B). Subsequently, the COVID-19 NP solution reference material (GBW(E)091097) was diluted into a series of standard solutions (1.0, 0.1, 10^−2^, 10^−3^, 10^−4^, 10^−5^, 10^−6^, and 0 μg/mL) with lysis buffer and then detected by the LFIA test strips assembled with the optimized parameters. The results are shown in Figure 5C: the signal intensity detected by the naked eye increased with the concentration increasing from 0.01 μg/mL to 1.0 μg/mL, and only a weak red band was presented on the test line when the concentration was 0.01 μg/mL, which indicates that the detection sensitivity was 0.01 μg/mL. However, the signal intensity on the test lines was enhanced after copper deposition-induced signal amplification, and a weak band was also developed in 0.01 ng/mL; the results demonstrate that the sensitivity was increased by three orders of magnitude after copper deposition. The detection limit of the developed GCNP-LFIA for the SARS-CoV-2 NP antigen was 10 pg/mL. Because the sample volume used for detection was only 10 μL, the developed method has the capability of detecting 0.1 pg NP antigen within 20 min.

The specificity of the proposed GCNP-LFIA was evaluated using the recombinant proteins of SARS-CoV and Middle East respiratory syndrome coronavirus. GCNP-LFIA had a cross-reactivity with SARS-CoV because of the high homology between SARS-CoV-2 and SARS-CoV (Figure S3). However, almost no SARS-CoV infection has been reported at present; therefore, the accuracy of the results obtained by GCNP-LFIA is considered reliable. As shown in Table 1, the sensitivity of the proposed LFIA is not superior to the previously reported LFIAs. However, the results prove the feasibility of copper deposition signal enhancement technology for GCNP-based LFIAs, which exhibit broad prospects for applications and social benefits. As for the SARS-CoV-2 detection, the LOD of the proposed detection method was lower, and the virus infection could be found earlier; thus, its detection capability needs to be improved in our further work. Additionally, the limitation of this work is the lack of evaluation of cultured virus or real samples, although the detection of the NP antigen in nasopharyngeal swab samples was expected. Nonetheless, the imperfection of this work does not obscure its implication for copper deposition signal enhancement. 

In this work, a simple and portable device was designed to allow the copper deposition reaction to take place in an independent space for each LFIA strip, which can simplify the operational process and improve the reaction efficiency. This work extensively applied the GCNP-mediated copper deposition signal amplification to the LFIA in accordance with the research achievements of Liu’s group [22]. The results indicated that the GCNP-based LFIA combined with copper deposition is worthy of popularization and application in COVID-19 detection and even in other areas.

## 4. Conclusions

This study developed a GCNP-based LFIA for sensitive SARS-CoV-2 NP antigen detection and proved that a GCNP-mediated copper deposition technique further enhances the LFIA signal. Under the optimized parameters, the detection limit of the developed GCNP-LFIA coupled with copper deposition for SARS-CoV-2 NP antigen detection was 10 pg/mL, which indicates a better sensitivity than the reported GCNP-LFIA. It provides a convenient supplementary approach to rapidly screen the suspected population for the prevention and control of the COVID-19 pandemic. However, further investigation with real samples is warranted.

## Figures and Tables

**Table 1:** Comparison of the performances among the developed method and other reported methods for SARS-CoV-2 NP antigen detection.

Method	Sensitivity	Time	Reference
Colloidal gold nanoparticle based LFIA	250 pg/mL	15 min	Mertens et al. [26]
Half-strip lateral flow assay	650 pg/mL	about 20 min	BD Grant et al. [12]
Cellulose nanobead-based LFIA	20 ng/mL	20 min	Kim et al. [14]
Fluorescent immunochromatographic assay	/	10 min	Diao et al. [15]
Fluorescent immunochromatographic assay based on multilayer quantum dot nanobeads	5.0 pg/mL	15 min	Wang et al. [18]
Dual-Mode fluorescence lateral flow immunoassay	0.5 pg/mL	10 min	Wang et al. [19]
Cotton-tipped electrochemical immunosensor	0.8 pg/mL	about 20 min	Shimaa Eissa and Mohammed Zourob [9,10]
Parallel reaction monitoring mass spectrometry assay	2 × 10^5^ viral particles/mL	about 3 h	Cazares et al. [9]
Colloidal gold nanoparticles based LFIA with copper deposition	10 pg/mL	Within 20 min	This work

## Data Availability

The authors confirm that the data supporting the findings of this study are available within the article.

## Supplementary Materials

The following supporting information can be downloaded at: https://www.mdpi.com/article/10.3390/bios12010013/s1, Figure S1: The result of antibody pair optimization; Figure S2: UV-vis spectra of the GCNPs and GCNP-antibody I detection probes; Figure S3: Evaluation of the specificity of the proposed GCNP-LFIA.
