# Supplementary material for: Lateral Flow Immunoassay Coupled with Copper Enhancement for Rapid and Sensitive SARS-CoV-2 Nucleocapsid Protein Detection
Source: Biosensors (Basel). 2021 Dec 29;12(1):13. doi: 10.3390/bios12010013 (PMC8773578; doi:10.3390/bios12010013)
Supplement: Supplementary file 1 [file biosensors-12-00013-s001.zip › biosensors-1488943-supplementary.pdf]

Supplemental Information

Lateral Flow Immunoassay Coupled with Copper Enhancement for Rapid and Sensitive SARS-CoV-2 Nucleocapsid Protein Detection

Tao Peng [1], Xueshima Jiao [1,2], Zhanwei Liang [1,2], Hongwei Zhao [3], Yang Zhao [1], Jie Xie [1], You Jiang [1], Xiaoping Yu [2], Xiang Fang [1] and Xinhua Dai [1,*]

1 Technology Innovation Center of Mass Spectrometry for State Market Regulation, Center for Advanced Measurement Science, National Institute of Metrology, Beijing 100029, China; pengtao@nim.ac.cn (T.P.); s20090710020@cjlu.edu.cn (X.J.); s20090710033@cjlu.edu.cn (Z.L.); zhaoy@nim.ac.cn (Y.Z.); xiejie@nim.ac.cn (J.X.); jiangyou@nim.ac.cn (Y.J.); fangxaing@nim.ac.cn (X.F.)
2 College of Life Sciences, China Jiliang University, Hangzhou 310018, China; yxp@cjlu.edu.cn
3 College of Ecology and Environment, Hainan University, Haikou 570228, China; hwzhao@hainanu.edu.cn
* Correspondence: daixh@nim.ac.cn; Tel.: +86-010-6452-4962; Fax: +86-010-6452-4962

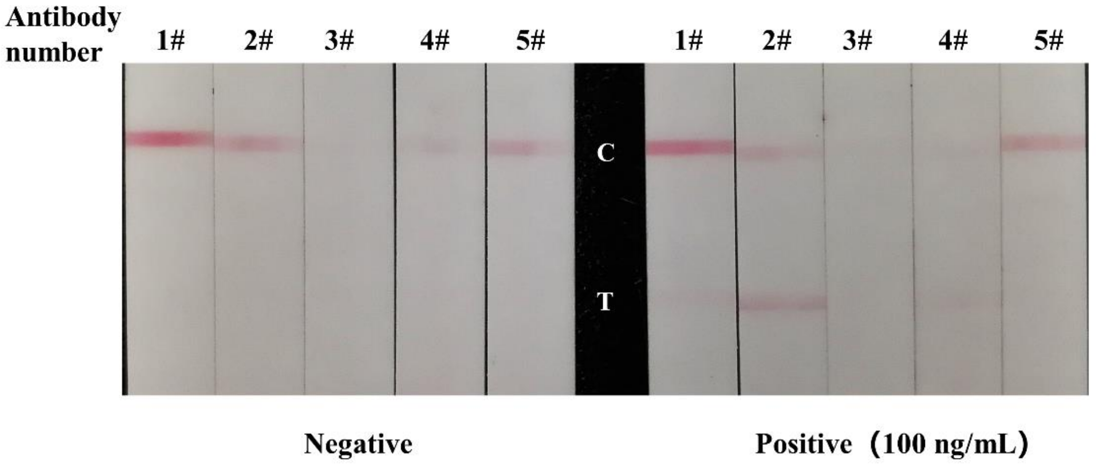

Figure S1. The result of antibody pair optimization.

Six anti-NP antibodies were obtained from companies and numbered as 1#, 2#, 3#, 4#, 5#, 6#, which were primarily evaluated by the lateral flow immunoassay and some antibodies were screened. According to our primarily results, then 6# antibody was coated on the NC membrane, and 1-5# antibodies were conjugated with the GCNPs. The result indicated that 2# and 6# antibody were selected as the detection antibody and capture antibody, respectively.

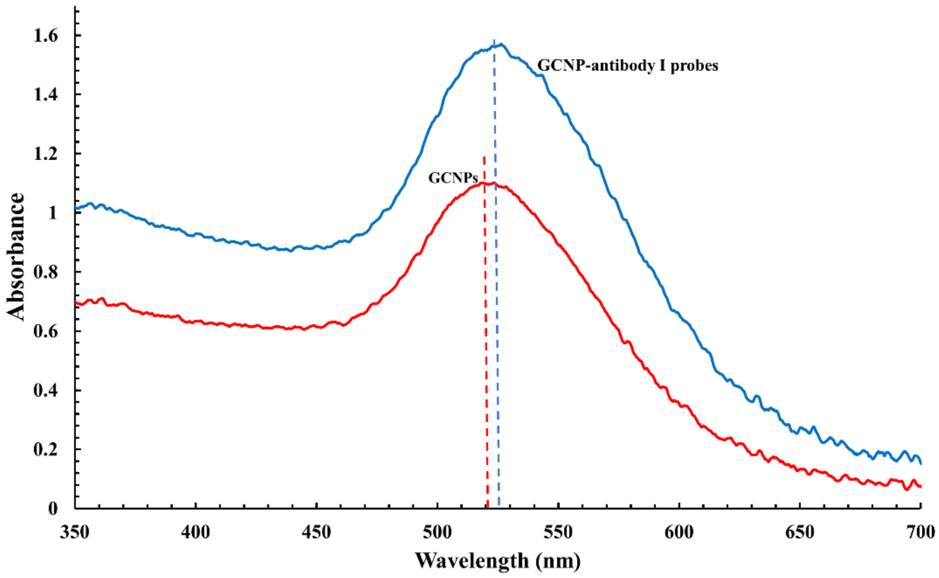

Figure S2. UV-vis spectra of the GCNPs and GCNP-antibody I detection probes.

The UV-vis spectra were obtained by Nanodrop One[c] (Thermo Fisher Scientific). It indicated that the characteristic absorption peak red shifted (about from 521 nm to 523 nm), which demonstrated that the antibody I conjugated with GCNPs successfully.

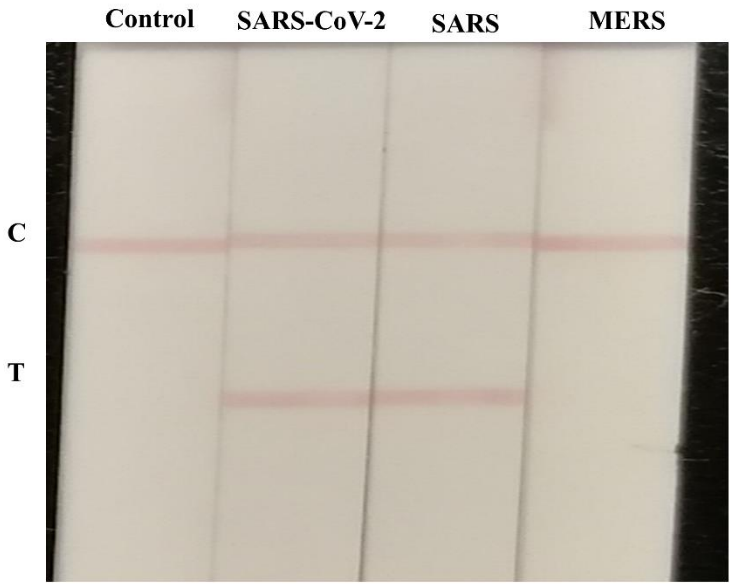

Figure S3. Evaluation of the specificity of the proposed GCNP-LFIA.

The specificity of the proposed GCNP-LFIA has been evaluated with the recombinant protein of SARS and MERS, which indicated that it has high cross-reactivity with SARS, because SARS-CoV-2 and SARS virus have high homology.
